# Horizon scan of the clinical development landscape of biosimilar products in the UK and EU

**DOI:** 10.1017/S0266462326103468

**Published:** 2026-01-26

**Authors:** Amy Hussain, Ross Fairbairn, Sarah Khalid Khan

**Affiliations:** Population Health Sciences Institute, Newcastle University Faculty of Medical Sciences, Newcastle upon Tyne, UK

**Keywords:** horizon scanning, biosimilars, medicines, emerging technologies, pharmaceutical innovation

## Abstract

**Objectives:**

Biosimilars are biological medicines highly similar to an authorized reference medicine, offering substantial cost savings and increased treatment access. Despite the regulatory framework in the UK and EU facilitating their approval, the biosimilar landscape remains small compared to small-molecule drugs. This study provides a horizon scanning overview of the current biosimilar landscape, procured through horizon scanning activities.

**Methods:**

Data were sourced from ClinicalTrials.gov and the EU Clinical Trials Register, scanned monthly to identify innovative medicines in clinical development. We included biosimilars identified through horizon scanning from April 2017 to February 2025. Supplementary data were collected from the European Medicines Agency to ascertain approval status, and additional clinical trial information was manually extracted from relevant registries.

**Results:**

We identified 156 unique biosimilars developed across 174 clinical trials, with sixty-four approved by the MHRA and seventy-eight by the European Medicines Agency. Adalimumab, bevacizumab, and denosumab were the reference products with the most biosimilars in development. Most biosimilar trials were at phase III. There are seventy-one biosimilars in active development.

**Conclusions:**

The development landscape of biosimilars in the UK and EU show high activity levels. Continuous improvements in horizon scanning methods and regulatory frameworks are essential to support the timely adoption of biosimilars, maximizing their benefits for healthcare systems.

## Introduction

A biosimilar, as defined by the European Medicines Agency (EMA), is a biological medicine that is highly similar to an already-authorized biological medicine, known as the reference medicine. Biological medicines have an active substance derived from a biological source, such as living cells or organisms (1).

A biosimilar must be proven to be similar enough to a reference product, using criteria such as structure, biological activity, safety, efficacy, and immunogenicity, to be granted approval (1). Pharmaceutical companies must wait until the period of market protection given to the reference medicine has expired before they can market a biosimilar (2). Within several years, biological medicines lose the patent protection, but developing new medicines costs a significant amount of both time and money. This expense poses a substantial economic burden for providers and healthcare systems, and in the UK, this can lead to significant costs for the taxpayer or disinvestment in other health resources (3;4). Biosimilars offer substantial savings and increased treatment access compared to their reference products due to their lower development costs. The comprehensive comparability exercise required for approval reduces the need for extensive preclinical and clinical trials, saving time and resources (5). By fostering a competitive pharmaceutical market, biosimilars drive innovation and lower prices, benefiting patients and healthcare systems (6). Biosimilars occupy a comparable regulatory and market position to generics, in that both are follow-on products developed after the patent expiry of reference medicines. However, whereas generics relate to small-molecule drugs and are exact chemical copies, biosimilars apply to biological medicines and are highly similar but not identical to their reference products (7).

In the United Kingdom (UK), the use of biosimilars has become quite prominent in recent years, with estimates of 86–90 percent for the uptake of biosimilars for top-selling reference medicines like rituximab and trastuzumab. This has allowed the National Health Service (NHS) to make savings of more than £200 million by switching to biosimilar medicine use in 2017 and 2018 (8). These figures are projected to increase to £300 million of savings per year for the NHS in the future (9). Similar progress has also been made across Europe, with biosimilar uptake continuing a steady rise compared to overall market share, albeit with fragmented benefits across different European countries. It is estimated that biosimilar competition has saved €56 billion across Europe as of July 2024 (10–12).

In 2004, the EU established a robust regulatory framework for biosimilar approval, requiring all biotechnology-derived medicines, including biosimilars, to undergo evaluation via the centralized procedure in the EMA. Most biosimilars approved have followed this pathway. When a company submits a biosimilar marketing authorization application to the EMA, it undergoes evaluation by various bodies including the Committee for Medicinal Products for Human Use (CHMP) and the Pharmacovigilance Risk Assessment Committee, EU experts on biological medicines (Biologics Working Party) and specialists in biosimilars (Biosimilar Working Party). This assessment culminates in an EMA scientific opinion, forwarded to the European Commission, which, upon positive CHMP feedback, grants EU-wide marketing authorization (MA) (2).

The very first biosimilar medicine, Omnitrope (a biosimilar for the growth hormone somatropin) was approved in 2006 in the EU (13). As of March 2025, 102 biosimilars have central MAs for the EU by the EMA (14).

Following the UK’s departure from the EU (Brexit), the Medicines and Healthcare products Regulatory Agency (MHRA) took over biosimilar medicine regulation within the UK starting 1 January 2021 (15). In May 2021, the MHRA issued new guidance for biosimilar approval, which, although based on CHMP guidelines, introduced some differences from the original EMA guidance. The new MHRA process simplifies approval and aims for a more streamlined approach, reducing the need for extensive trial testing. Notably, comparative efficacy trials may be waived if supported by scientific rationale, contrasting with EMA guidelines mandating such trials for approval (2;16).

In England and Wales, the National Institute for Health and Care Excellence (NICE) evaluates medicines by assessing clinical and economic evidence submitted by various stakeholders. This evaluation determines whether a medicine should be recommended as a clinically effective and cost-effective use of NHS resources, and for whom it should be available. A positive appraisal results in NICE guidance, which is crucial for accessing the UK market, as the NHS is required by law to provide medicines recommended by NICE (17;18). Previously, NICE evaluated biosimilar medicines when notified by the National Institute for Health and Care Research (NIHR) Innovation Observatory (IO) for technology appraisal (TA) (19). However, the process has since evolved, and NICE has determined that biosimilars do not need an individual TA, as any positive TA recommendation(s) that exists for the reference product also applies to the biosimilar, since they contain a version of the same active substance (20). Consequently, all guidance applicable to the reference drug also applies to the biosimilar once available in the NHS. NICE may issue an evidence summary if further review is warranted (19). To date, NICE has produced three biosimilar evidence summaries (21).

Following the adoption of a regulatory framework for the approval of biosimilars in the EU, other countries in Latin America (22) and Asia Pacific (23;24) have created their own frameworks, which has increased the uptake of biosimilars globally. It is evident that biosimilars play a crucial role in healthcare systems, given their potential to provide significant cost savings and increase treatment access, thus there is a growing interest in monitoring their development and approval. Horizon scanning has an important role to play in facilitating the uptake of biosimilars, by providing early intelligence on biosimilars in development for health systems, allowing for timely health technology assessments (HTA) and improving regulatory responses to shifting trends.

The IO has been conducting horizon scanning activities to capture innovative medicinal products in clinical development since 2017, including that of biosimilars, on our internal database known as the Medicines Innovation Database (MInD). This work is carried out to support the activities of national health and care decision makers in England, including topic selection and prioritization activities for NICE health technology programs and various NHS England groups under the Innovation, Research and Life Sciences and Strategy group. The MInD serves as a vital tool for tracking biosimilar development pipeline, aligning with our broader aim to provide insights into the current biosimilar landscape and future approvals. By addressing key questions such as the number of biosimilars in development, leading pharmaceutical companies, development stages, approval status, and unique characteristics of clinical trials, we aim to contribute valuable information to stakeholders involved in biosimilar pharmaceutical development, healthcare decision-making, and policy development.

## Aims and objectives

To provide an overview of the current landscape of biosimilars, as well as exploring potential future biosimilar approvals. The key questions this paper will address are:

- What are the characteristics of the biosimilars currently in clinical trials in the UK and EU?
- Which biosimilar developers are currently leading the market in the UK and EU
- What are the biosimilars that are approved for reference products in the UK and EU?

## Methods

### Data sources

Data are on biosimilars in clinical trials is held on MInD and sourced from ClinicalTrials.gov and the EU Clinical Trials Register. These registries are scanned monthly for innovative medicines being tested in clinical trials at phase 1/2 and beyond (with some phase 1 trials added on an ad-hoc basis) and with locations in the UK, EU, US, Canada, or Australia. Searches of the two clinical trial registries are exported monthly and the resulting trials are analyzed by a researcher to determine whether a new entry to MInD is to be created. This decision is based on complex horizon scanning criteria which allows the IO team to provide relevant information for HTA stakeholders in England. All types of innovative medicines are included in MInD except for dietary supplements, prophylactic vaccines, and generic medicines (25).

Data on biosimilar medicines captured in MInD are combined with a regular biosimilar scan to maintain a bespoke biosimilar data set, which feeds an interactive dashboard hosted on the IO website (https://www.io.nihr.ac.uk/dashboardpages/biosimilar-medicines-monitored-by-the-nihr-innovation-observatory/). The biosimilar scan is conducted to capture biosimilars that have an MA from the MHRA or EMA but do not have clinical trials previously captured by the IO. New MHRA and EMA approvals are scanned monthly to capture any biosimilar approvals that are the result of regulatory submissions that do not include evidence directly from clinical trials and have therefore not been included on MInD previously. Generic medicines are not in scope for this analysis.

A snapshot of this data set was extracted and analyzed in February 2025. The fields extracted included intervention name, intervention aliases, condition, therapeutic area, primary developer, and clinical trial ID. For biosimilars that were linked to two or more clinical trials, one of the trials was designated as the “Main” trial, based on trial phase and number of participants.

### Supplementary data collection

Supplementary information for the biosimilars was collected and added to the data set. Regulatory approval data for biosimilars were collected from the EMA medicines register, the MHRA products website, or the Electronic Medicines Compendium (February 2025) to ascertain the approval status of biosimilars in the EU or UK. Administrative clinical trial information not available in MInD (trial phase, trial start date, and primary completion date) was extracted manually from ClinicalTrials.gov and EU Clinical Trial Register entries (February 2025).

### Data analysis

The data set included biosimilars identified through active horizon scanning from April 2017 to February 2025. Following extraction of the data, each record was reviewed by a researcher (AH or RF) AH or RF to confirm the inclusion of the record to a final data set. Where multiple clinical trials were captured for a single clinical trial, a researcher (AH or RF) identified the trial with the highest phase of development as the “main trial,” which was prioritized for analysis to allow for clearer conclusions to be drawn. This selection was made based on the trial phase, size, locations, and outcomes. Analyses and visualization creation were carried out using Microsoft Excel.

## Results

### Biosimilars identified in horizon scanning

Following the application of the sifting and review process, we identified 156 unique biosimilar interventions in 174 (140 main trials) clinical trials since April 2017, belonging to 31 reference medicines (Supplementary Table 1). Denosumab, bevacizumab, and adalimumab were the reference products for which the most biosimilars were identified (*n* = 14, *n* = 13, and *n* = 12, respectively), whilst only one biosimilar was identified for six different reference products.

**Figure 1.**
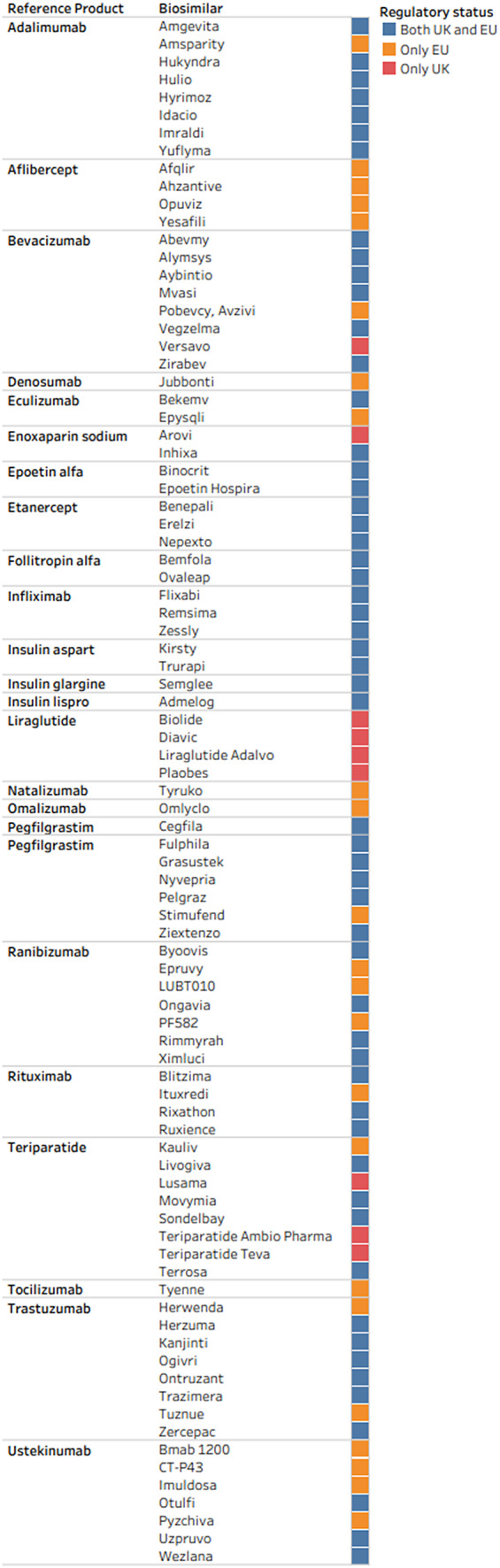
Regulatory status of biosimilars in the UK and EU.

Sixty-four (41 percent) of 156 biosimilars are currently approved by the MHRA for use in the UK and seventy-eight (50 percent) are currently approved by the EMA for use in the EU, as shown in Figure 1.

**Figure 2.**
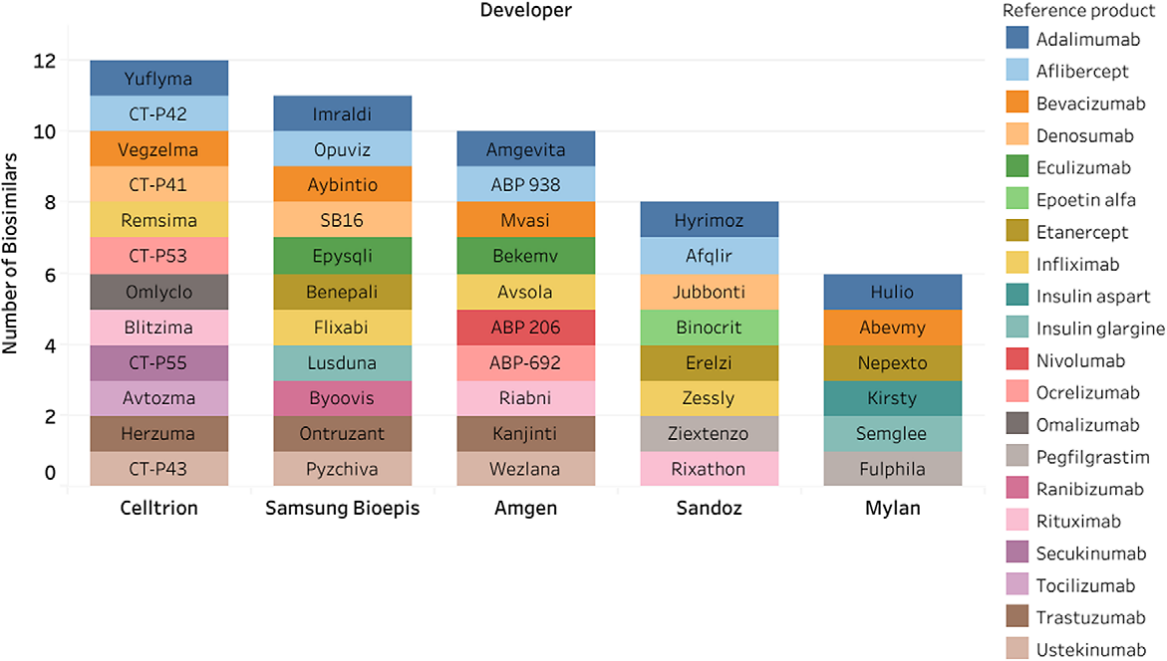
Top five developers for biosimilars.

For the 156 identified biosimilars, there were seventy-four unique developers. Celltrion, Samsung Bioepis, and Amgen were the listed developer with the most biosimilars (*n* = 12, *n* = 11, and *n* = 10, respectively) (Figure 2).

The reference products for the 156 biosimilars often targeted several conditions; however, analysis found that majority of the biosimilars were indicated exclusively for non-oncology conditions (*n* = 118, 75 percent), and 31 (20 percent) biosimilars were for exclusively for non-oncology conditions, whilst 8 (5 percent) were indicated for both oncology and non-oncology conditions.

### Biosimilar clinical trials

A total of 174 clinical trials were identified using horizon scanning methods. Of the 156 biosimilars identified, twenty-eight were studied in more than one clinical trial. For these twenty-eight biosimilars, one of the clinical trials was prioritized as a main trial for analysis. Sixteen biosimilars did not have a publicly available clinical trial listing at the point of data collection.

**Figure 3.**
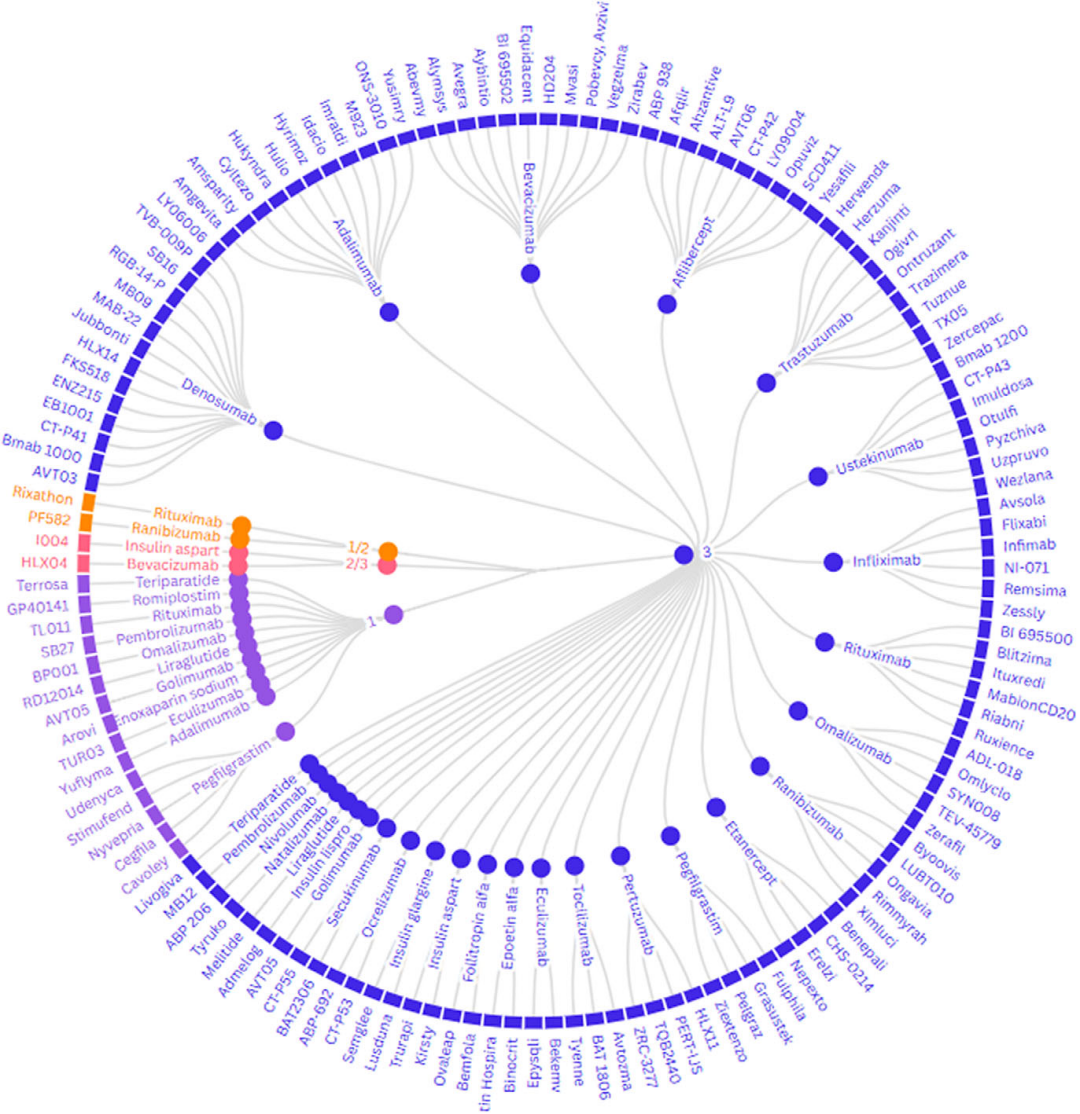
Clinical trial phases for biosimilars in development.

The main clinical trials for unique biosimilars identified were conducted as phase 3 trials (*n* = 120, 87 percent). Fifteen (11 percent) main trials were conducted at phase 1, two (1 percent) were phase 1/2 trials, and two (1 percent) trials were a phase 2/3 trial (Figure 3).

**Figure 4.**
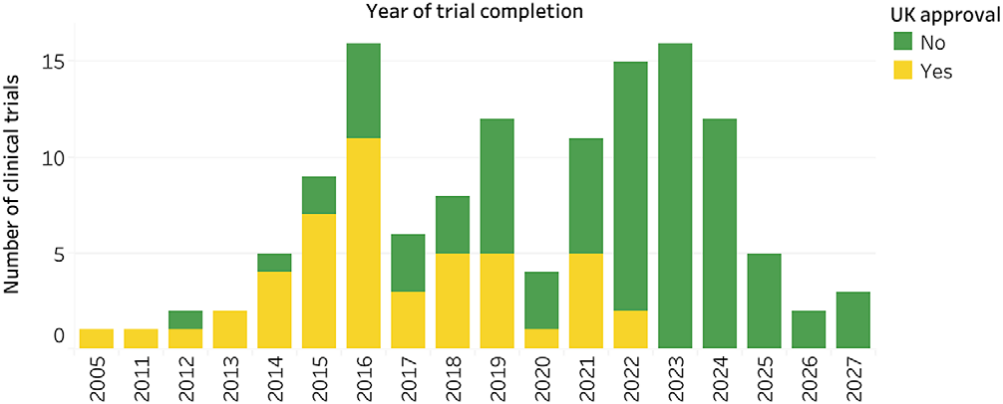
Primary completion dates by year of main clinical trials for biosimilars.

Primary completion dates were available for 140 of the main clinical trials for biosimilars. The most common actual or estimated years for trials to complete were 2023 (*n* = 16 trials), 2022, and 2016 (*n* = 16 trials) (Figure 4).

### Biosimilars in active development

Of the ninety-two biosimilars identified that were not approved by the MHRA, seventy-one were classified as being in active clinical development (Table 1). Of these interventions, biosimilars for the reference product denosumab had the highest development activity (*n* = 14 biosimilars), followed by biosimilars for aflibercept (*n* = 10) and omalizumab (*n* = 6).

**Table 1.** Unapproved biosimilars in the UK, considered to be in active development

Reference product	Biosimilar	Developer	Year of trial completion
Adalimumab	Yusimry	Coherus Biosciences	2016
Aflibercept	ABP 938	Amgen	2022
	Afqlir	Sandoz	2022
	Ahzantive	Formycon AG	2022
	ALT-L9	Altos Biologics	Unknown
	AVT06	Alvotech	2023
	CT-P42	Celltrion	2022
	LY09004	Luye Pharma	2023
	Opuviz	Samsung Bioepis	2021
	SCD411	Sam Chun Dang Pharm	2022
	Yesafili	Biocon	2020
Bevacizumab	Avegra	Biocad	2014
	HD204	Prestige Biopharma	2024
	HLX04	Shanghai Henlius Biotech	2025
	Pobevcy, Avzivi	Bio-Thera Solutions	2019
Denosumab	AVT03	Alvotech	2024
	Bmab 1000	Biocon	2024
	CT-P41	Celltrion	2023
	EB1001	Eden Biologics	Unknown
	ENZ215	Enzene Biosciences	2024
	FKS518	Fresenius Kabi	2023
	HLX14	Shanghai Henlius Biotech	2023
	Jubbonti	Sandoz	2022
	LY06006	Luye Pharma	2025
	MAB–22	Xentria	2027
	MB09	mAbxience	2023
	RGB–14-P	Gedeon Richter	2023
	SB16	Samsung Bioepis	2022
	TVB–009P	Teva Pharmaceuticals	2022
Eculizumab	Epysqli	Samsung Bioepis	2021
	TUR03	Turgut Ardika Pty Ltd	2024
Filgrastim	Zefylti	CuraTeQ Biologics	N/A
Golimumab	AVT05	Alvotech Swiss AG	2023/2024
Insulin aspart	I004	Amphastar Pharmaceuticals	2023
Liraglutide	Levim Liraglutide	Levim Biotech	N/A
	Lirafit	Glenmark	N/A
	Melitide	Cinnagen	2019
	RD12014	Sunshine Lake Pharma	2020
Natalizumab	Tyruko	Polpharma Biologics	2021
Nivolumab	ABP 206	Amgen	2025
Ocrelizumab	ABP–692	Amgen	2027
	CT-P53	Celltrion	2027
Omalizumab	ADL–018	Kashiv BioSciences	2024
	BP001	Alvotech	2019
	Omlyclo	Celltrion	2022
	SYN008	CSPC Baike Biopharmaceutical	2023
	TEV–45779	Teva Pharmaceuticals	2024
	Zerafil	CinnaGen	2023
Pembrolizumab	MB12	Laboratorio Elea	2026
	SB27	Samsung Bioepis UK Ltd	2025
Pertuzumab	HLX11	Shanghai Henlius Biotech	2024
	PERT-IJS	Biocon	2026
	TQB2440	Chia Tai Tianqing Pharmaceutical Group	2023
	ZRC–3277	Zydus Lifesciences	2023
Ranibizumab	LUBT010	Lupin Pharmaceuticals	2024
	PF582	Pfenex	2016
Rituximab	MabionCD20	Mabion	2023
	Riabni	Amgen	2019
Romiplostim	GP40141	Geropharm	2024
Secukinumab	BAT2306	Bio-Thera Solutions	2024
	CT-P55	Celltrion	2025
Tocilizumab	Avtozma	Celltrion	2023
	BAT 1806	Bio-Thera Solutions	2021
	Tyenne	Fresenius Kabi	2021
Trastuzumab	Tuznue	Prestige Biopharma	2019
	TX05	Tanvex BioPharma	2020
Ustekinumab	Bmab 1200	Biocon	2023
	CT-P43	Celltrion	2021
	Imuldosa	Accord Healthcare	2022
	Pyzchiva	Samsung Bioepis	2022

Analysis of the developers of the seventy-one biosimilars listed which are unlicensed and considered to be in active development (Table 1) found that Celltrion was the most active developer, developing seven biosimilars. Biocon was actively developing five biosimilars, followed by Samsung Bioepis, Amgen, and Alvotech developing four biosimilars each. Altogether, there were fifty-five unique developers with unlicensed biosimilars in active development.

## Discussion

### Biosimilar horizon scanning landscape

The relatively small size of the biosimilar pipeline reflects the more recent emergence and smaller market share of biologics compared to small-molecule medicines, which still account for around 90 percent of global drug sales (26). As biosimilars can only be approved once reference biologics have been authorized and their market exclusivities have expired, their growth is inherently tied to the earlier expansion of the biologics market. In 2024, biologics consisted of 41 percent of pharmaceutical expenditure by the European Union (10). On ending of exclusivity of these biologics, they will be open to the off-patent market where biosimilar development can improve access. Consequently, the biosimilar landscape can only grow at a rate analogous to the previous development of biological reference products and the market exclusivities and patent protections afforded to them.

The acceptance and uptake of biosimilars within healthcare systems also significantly influence their development. There are clear incentives for healthcare systems to transition patients from reference products to biosimilars, including often substantial cost savings for health systems when prescribing a biosimilar compared to a reference biologic, or improved patient access for treatments once deemed not cost-effective. However, initial concerns about safety, tolerability, and efficacy can hinder the transition from reference biologic to biosimilar prescribing (27). Significant progress has been made to facilitate such transitions, with the MHRA, EMA, and FDA releasing guidance allowing biosimilars to be freely interchangeable, albeit with some restrictions on pharmacy-level switching in certain regions (16;28;29). To further expand the biosimilar landscape, these positive steps must be continuously built upon both in the UK and globally. The utilization of real-world evidence (RWE) can play an important role in decision-making. RWE can inform health systems on patient preference, real-world biosimilar safety and efficacy, and clinical adoption, enabling the creation of stronger guidance and frameworks to aid biosimilar uptake (30).

However, barriers such as physician and patient skepticism, limited awareness, and the lack of robust education on biosimilars continue to pose significant challenges. Studies have shown that these barriers can be attributed to concerns over immunogenicity and the belief that biosimilars might not perform identically to their reference products in all patients. For instance, a study highlighted that many physicians are still not fully confident in prescribing biosimilars due to concerns about their efficacy and safety, leading to hesitation in switching patients from originator biologics to biosimilars (31;32). In regions where skepticism persists, local regulators or clinicians prefer safety and efficacy data generated within their own context. Adoption patterns vary significantly across countries, with those producing reference biologics or biosimilars often demonstrating higher confidence and faster uptake, whereas non-producing countries may require additional local data or context-specific evaluations. These contextual differences emphasize the need for region-sensitive evidence strategies and early identification of markets where supplementary RWE may be necessary to overcome adoption barriers (33). Additionally, the “nocebo effect,” where negative expectations about biosimilars lead to poorer outcomes, further exacerbates these issues and underscores the need for comprehensive education efforts (31). Moreover, financial incentives for prescribing reference biologics over biosimilars, as well as contractual arrangements with manufacturers of original biologics have been identified internationally, which can further impede the uptake of biosimilars. Evidence suggests that the lack of financial incentives and the administrative burden associated with obtaining prior authorizations for biosimilars contribute to their slower-than-expected adoption rates (34;35). In some cases, health providers prefer originator biologics due to established rebate agreements, which can also dissuade prescribers from switching to biosimilars (35;36).

A global study that included the UK, Southern and Northern American, the EU, and Asian countries in its analysis found that, following the introduction of a biosimilar onto the market, average prices per dose of pertinent reference medicines were immediately reduced. For example, most notably, trastuzumab biologics price observed an immediate price reduction of 27.7 percent and a decrease over time by 484.2 percent per year, post-biosimilar entry. Although this highlights the financial benefit of introducing biosimilars in the market, these price reductions may contribute to limited uptake of biosimilars globally (37). The UK and EU face less of these challenges, as frameworks exist to prescribe biosimilars for treatment naïve patients (38). However, it has been found that the biosimilar markets in European countries are influenced by highly heterogenous adoption and substitution measures, leading to calls for standardized guidance to be created (39).

The pharmaceutical industry demonstrates high interest in biosimilars, as evidenced by the seventy-four companies actively developing these products. Notably, Celltrion and Samsung Bioepis were identified as the most active developers. This interest is further reflected in the creation of unique biosimilar brands or organizations by large, established pharmaceutical companies. For instance, Amgen has created a separate entity, Amgen Biosimilars, and Merck in 2021 planned to spin off its biosimilar pipeline to a partner company, Organon (40;41). Additionally, Sandoz, the generic pharmaceuticals and biosimilars spin-off division of Novartis, launched the “Act4Biosimilars” initiative in 2022, aiming to increase patient access and reduce health inequality through biosimilars, by increasing global biosimilar adoption by at least thirty percentage points in over thirty countries by 2030 (42).

There were seventy-one biosimilars unlicensed in the UK, in active clinical development by fifty-five different developers. The analysis revealed that denosumab, aflibercept, and omalizumab are the reference products for which the most biosimilars are being actively developed. The heightened development activity for these products may correlate with their approaching or already approached market exclusivity expirations and significant market size, e.g. aflibercept has an estimated loss of market exclusivity in the EU in 2027, denosumab has a date of 2025 in the UK, whilst bevacizumab had a patent expire in 2019 (43–45). Conversely, reference products like infliximab and pegfilgrastim, which have several approved biosimilars, show less active development. Development windows for biosimilars are short as the first developers to enter the market often gain the larger market shares (46). As a result, entering a biosimilar to market late may not be an economically viable strategy for a developer.

The majority of the biosimilar clinical trials (87 percent) were conducted at phase 3, reflecting the nature of evidence generation required for biosimilar approval. Typically, only one randomized controlled trial is necessary to demonstrate equivalence with the reference product (47). Recent MHRA guidance indicates that in some instances, a comparative efficacy trial may not be required if supported by substantial scientific rationale (16). This approach aligns with positions adopted by other major regulators, including the EMA (48) and FDA (49), who have also recently evaluated the extent to which comparative efficacy trials are required for biosimilar submissions. This potential lack of available trials represents a challenge to horizon scanning systems and will require new methods to be developed to deliver timely intelligence. Horizon scanning organizations must therefore consider making changes to current processes and methods to accommodate a shorter timeline for new biosimilar products.

A notable limitation of this analysis is the lack of analysis of therapeutic conditions targeted by biosimilars, due to the difficulty arising in how biosimilar conditions are approved. Biosimilars typically mirror the broad range of indications held by their reference products, which are often licensed for multiple, distinct conditions. In principle, a biosimilar may eventually be approved for the full list of the reference product’s indications, even if clinical development initially focuses on a specific subgroup or population (2). For example, a rituximab biosimilar, CT-P10 was identified in clinical development for follicular lymphoma only (50). This biosimilar subsequently gained a license in the UK (51) and EU (52) (as Truxima and Blitzima) for multiple indications including non-Hodgkins lymphoma, chronic lymphocytic leukemia, rheumatoid arthritis, and more. These indications match the same listed ones as that of the reference product in the UK (53), illustrating how clinical trial activity often reflects only a subset of eventual licensed indications. As a result, attempts to categorize and analyze biosimilar development by therapeutic area or pathology risks being misleading or incomplete and were therefore not included in this paper. Addressing this challenge would require a mapping framework task between biosimilar trials and reference product indications, which was beyond the scope of this study.

### Future perspectives

The value of early awareness and abundance of information generation through horizon scanning of biosimilars is likely to increase in the future. With the sustained growth in the development of novel biologic medicines, an analogous increase in biosimilar development is expected (54). As regulatory pathways evolve, the clinical development of biosimilars is likely to become shorter and more efficient. Horizon scanning organizations should consider capturing information regarding the patent expiry and loss of exclusivity of biological reference products to anticipate new biosimilar introductions earlier than clinical trial tracking allows. In turn, this will allow for improved, earlier information to be communicated to national stakeholders who may rely on early awareness of new biosimilar products.

The activity in the field of biosimilar development from industry demonstrates clear interest in biosimilar products and their increasing importance for health care systems. It is important that regulators continue work to improve the acceptance and uptake of biosimilars to maximize the potential savings for health care systems. Initiatives such as the FDA Biosimilars Action Plan in the US and updated guidance and frameworks from the MHRA and NHS in the UK will aim to overcome these barriers in the future (55–57).

This project identified high levels of activity in the clinical development and licensing of biosimilar products in the UK and EU. This level of activity is expected to increase swiftly in the future due to increased interest in the area from industry, regulators, and health systems. Horizon scanning systems should expand the scope of their data collection and reporting to account for the unique challenges posed in the early identification of biosimilar products to aid in the decision making of HTA organizations.

## Supporting information

10.1017/S0266462326103468.sm001 Hussain et al. supplementary material Hussain et al. supplementary material

## Supplementary material

The supplementary material for this article can be found at http://doi.org/10.1017/S0266462326103468.
