# Supplementary material for: Horizon scan of the clinical development landscape of biosimilar products in the UK and EU
Source: Int J Technol Assess Health Care. 2026 Jan 26;42(1):e14. doi: 10.1017/S0266462326103468 (PMC12916240; doi:10.1017/S0266462326103468)

***Supplementary table 1. Unique biosimilars with reference products***


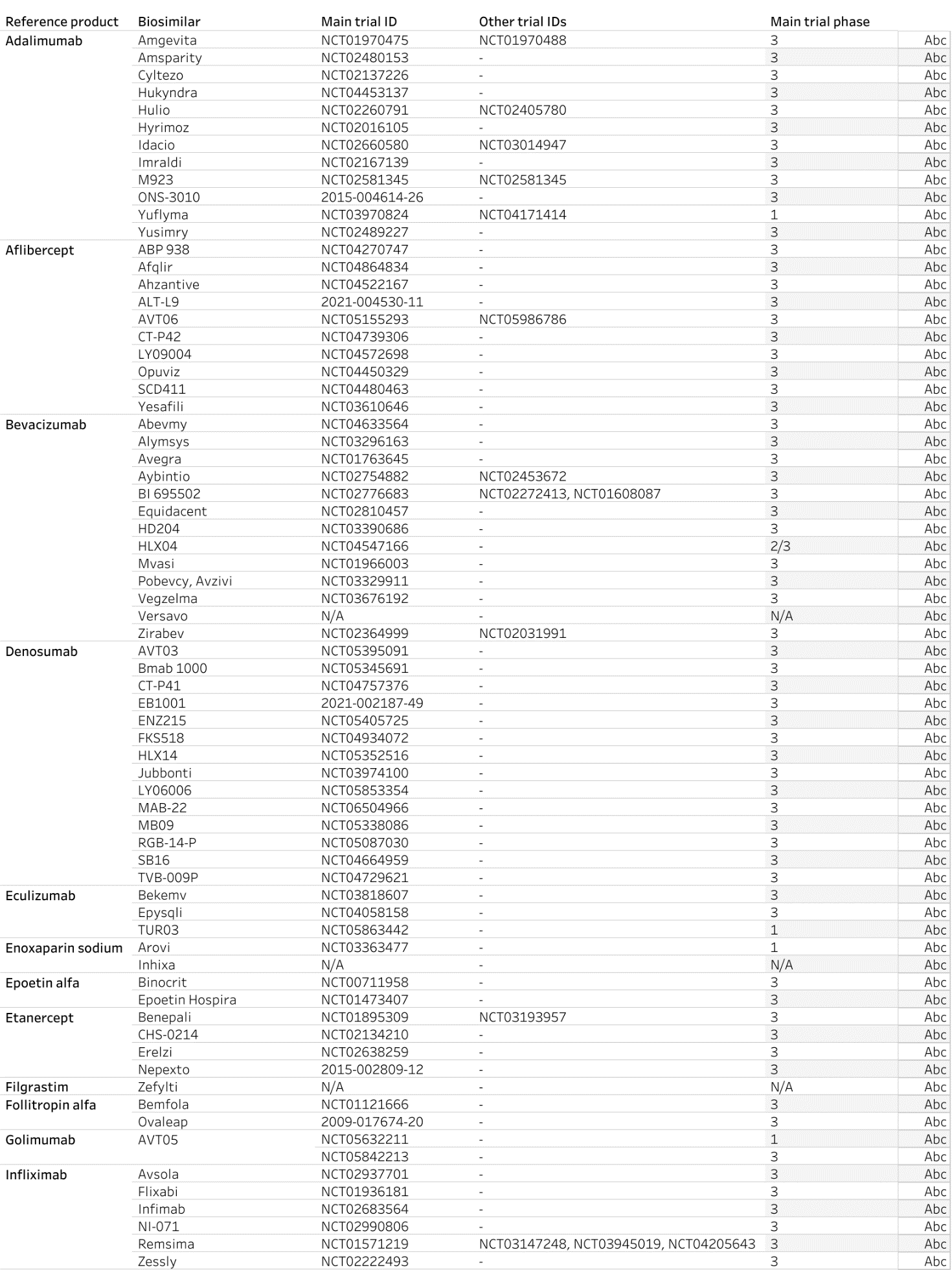


***Table 1 (continued). Unique biosimilars with reference products***


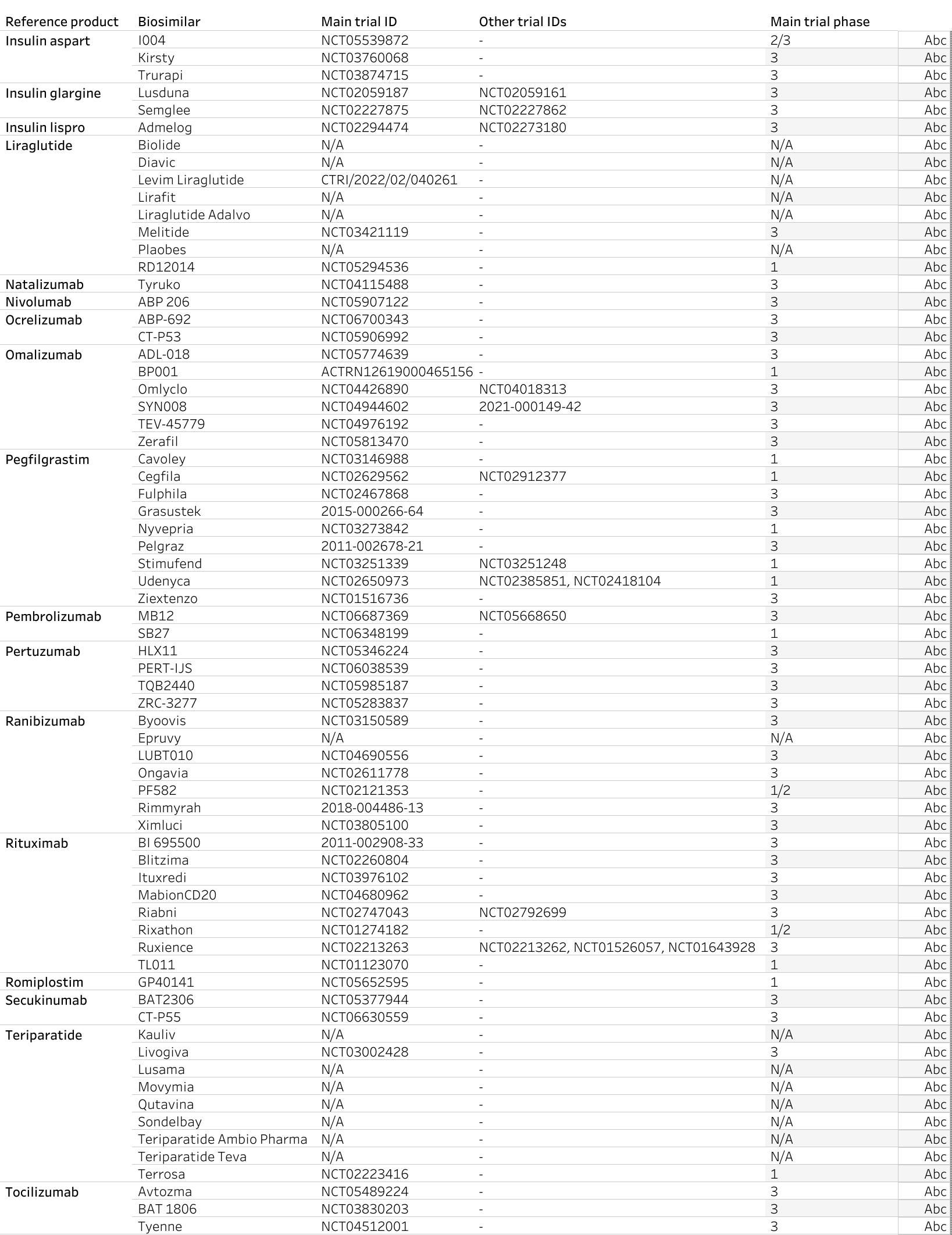


***Table 1 (continued). Unique biosimilars with reference products***
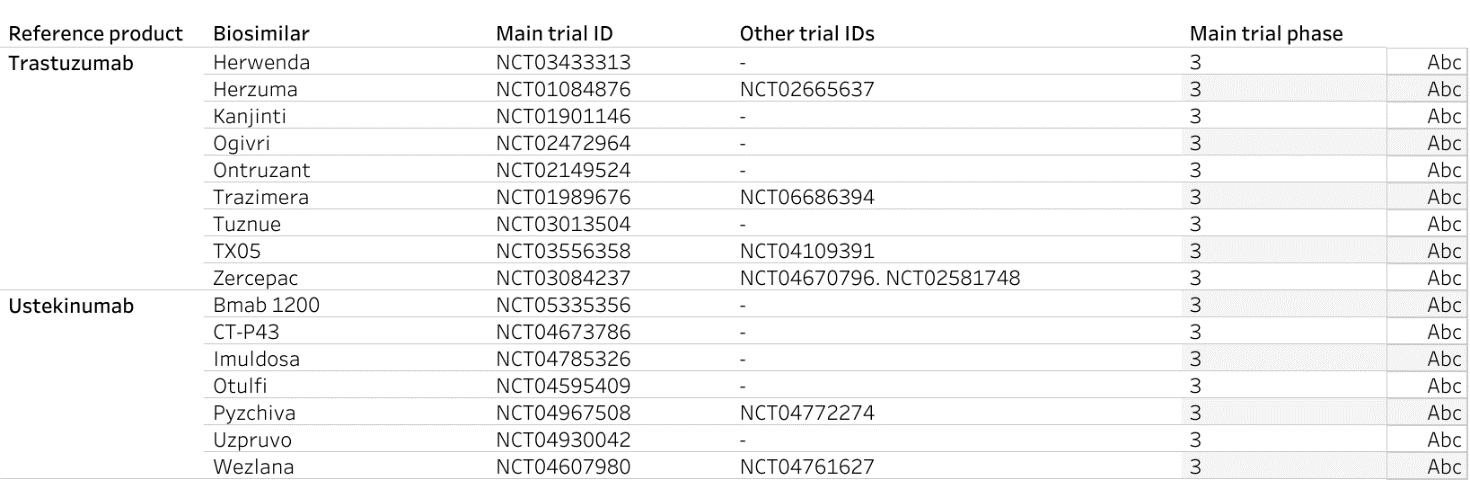

Supplement: Hussain et al. supplementary material [file S0266462326103468sup001.docx]
